# Sex-specific loss of mitochondrial membrane integrity in the auditory brainstem of a mouse model of Fragile X Syndrome

**DOI:** 10.1098/rsob.240384

**Published:** 2025-05-14

**Authors:** Claire Caron, Elizabeth Anne McCullagh, Giulia Bertolin

**Affiliations:** ^1^ CNRS IGDR (Institute of Genetics and Development of Rennes), University of Rennes, Rennes, F-35000 UMR 6290, France; ^2^ Integrative Biology, Oklahoma State University, Stillwater, OK, USA

**Keywords:** mitochondria, membrane integrity, auditory brainstem, medial nucleus of the trapezoid body, Fragile X Syndrome

## Abstract

Sound sensitivity is a common sensory complaint for people with autism spectrum disorder (ASD). How and why sounds are perceived as overwhelming by affected people is unknown. To process sound information properly, the brain requires high activity and fast processing, as seen in areas like the medial nucleus of the trapezoid body (MNTB) of the auditory brainstem. Recent work has shown dysfunction in mitochondria in a genetic model of ASD, Fragile X Syndrome (FXS). Whether mitochondrial functions are also altered in sound-processing neurons has not been characterized yet. To address this question, we imaged MNTB in a mouse model of FXS. We stained MNTB brain slices from wild-type and FXS mice with two mitochondrial markers, TOMM20 and PMPCB, located on the outer mitochondrial membrane and in the matrix, respectively. Our imaging reveals significant sex-specific differences between genotypes. Colocalization analyses between TOMM20 and PMPCB show that the integrity of mitochondrial subcompartments is most disrupted in female FXS mice compared with female wild-type mice. We highlight a quantitative fluorescence microscopy pipeline to monitor mitochondrial functions in the MNTB from control or FXS mice and provide four complementary readouts, paving the way to understanding how cellular mechanisms important to sound encoding are altered in ASD.

**Figure:**
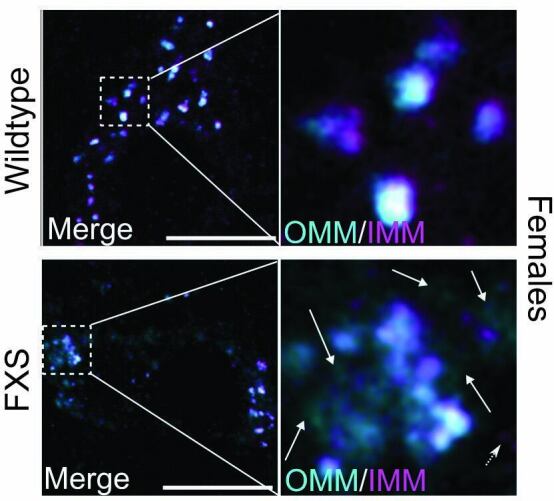

## 1. Introduction

Fragile X Syndrome (FXS) is the most common genetic cause of intellectual disability and autism spectrum disorder (ASD), occurring in 1 : 4000−1 : 8000 individuals [1], with prevalence being higher in males due to the X-linked nature of the disorder. FXS is caused by a CGG repeat expansion on the X chromosome in the Fragile X Messenger Ribonucleoprotein 1 *FMR1* gene, resulting in reduced or no expression of the protein fragile X messenger ribonucleoprotein (FMRP). FMRP has important functions in the nervous system, including regulating gene expression, and its loss results in symptoms including cognitive impairments, sensory issues—such as auditory sensitivity, anxiety and overall hyperactivity [2]. The role of FMRP in the functioning of the nervous system has grown to not just include repressing specific mRNAs but binding to RNA, proteins and channels resulting in the deficits seen in FXS [3]. Recent evidence also suggests that FMRP has roles in distinct functional pathways, including energy metabolism, which may underlie multiple aspects of the disorder. Notably, FXS patient fibroblasts showed doughnut-shaped mitochondria, excessive amounts of mitochondrial proteins and susceptibility to apoptotic cell death [4]. Other studies in a mouse model of FXS, *Fmr1* KO mice, showed an inner mitochondrial membrane (IMM) leak, altered morphology, impaired mitochondrial fusion in the hippocampus [5,6] and compartment-specific functional and morphological plasticity that is lost in FXS [7].

Mitochondria are multifunctional organelles that are essential for cell physiology and during stress response. They harbour the machinery devoted to energy production, the oxidative phosphorylation (OxPhos) complex, and they are key signalling hubs both internally and within cells. The wide range of metabolites produced by or transduced within mitochondria are important messengers not only for the different subcellular compartments but also for intercellular communication [8]. Interestingly, mitochondria as a whole are also a way for cells to communicate between them. Mitochondrial behaviours such as transport can be specialized in tissues within an organism to ensure specific cellular functions [9]. During typical mouse development, mitochondria are transferred between adjacent cells. This appears to be a multi-systemic feature occurring in several organs, including the brain [10]. In the brain, the transfer of mitochondria from astrocytes to neurons was shown to be neuroprotective after stroke [11]. This strongly indicates that energetically competent mitochondria are required at acute stress sites to overcome systemic failures.

Astrocyte functions were also shown to be impaired by a lower O_2_ availability and a lower mitochondrial energy production capacity in cultured astrocytes isolated from *Fmr1* KO mice [12]. While the overall mitochondrial ATP production levels were similar in male and female mice, this study showed that mitochondria from both wild-type and *Fmr1* KO male mice produced higher reactive oxygen species (ROS) than those in their respective female counterparts. Therefore, it is important to consider sex as another key variable, potentially determining mitochondrial specificity in different brain cells [13,14].

The auditory brainstem, and particularly the medial nucleus of the trapezoid body (MNTB), is emerging as a model for neuroenergetics due to the high energy demands required of this brain area to be able to reliably generate action potentials up to 1000 Hz [15]. Indeed, energy demands, as measured by O_2_ consumption, of this brain area increase up to 600 Hz, suggesting high energy demands that are dependent on temporal characteristics of stimulation [16]. In addition, mitochondrial volume increases during development in the MNTB, suggesting importance in increasing firing rates that occur during early developmental stages of auditory processing [17]. The auditory brainstem is required for sound localization processing, and the calyx of Held synapse, which is the presynaptic terminal onto MNTB cells originating from globular bushy cells in the cochlear nucleus, is used both as a model synapse and is critically important for sound location computation [18]. This is particularly relevant in regard to FXS, where auditory symptoms are common and some symptomatology may originate from early auditory brainstem function [19]. Specifically, research produced by ourselves and others has shown that deficits occur throughout the auditory system from the periphery through the entirety of the ascending auditory pathway [20–27]. These previous studies demonstrate several brainstem and midbrain-specific phenotypes including pronounced, yet subtle, deficits in auditory processing [28], imbalanced excitation and inhibition [20,21,29,30], synaptic deficits [31–33], high levels of FMRP [34], non-cortically induced audiogenic seizures [23,27] and myelination deficits [24,35].

A better understanding of the pathways involved in FXS is critical for developing therapies to remedy symptomology or rescue loss of FMRP. We hypothesize that mitochondrial functionality is critical to MNTB homeostasis and that the absence of FMRP leads to mitochondrial dysfunction. To this end, we explore the contribution of sex and the presence or absence of FMRP in mitochondria from MNTB neurons. In particular, we evaluate three complementary mitochondrial parameters, including the morphology of the mitochondrial network, mitochondrial integrity and mass. We uncover sex- and genotype-specific differences in mitochondrial integrity, and we provide evidence that these three mitochondrial parameters are correlated in MNTB neurons of FXS mice.

## 2. Methods

### 2.1. Ethical approval

All experiments complied with all applicable laws, National Institutes of Health guidelines and were approved by the Oklahoma State University IACUC, approval number 20-07. Investigators understand the ethical principles under which the journal operates and that their work complies with the animal ethics checklist.

### 2.2. Animals

Experiments were conducted in C57BL/6J (stock no. 000664, B6) wild-type background, hemizygous male and homozygous female *Fmr1* mutant mice (B6.129P2-*Fmr1^tm1Cgr^*/J stock no. 003025) obtained from the Jackson Laboratory (Bar Harbor, ME USA) and bred at Oklahoma State University [36]. Animals were generated for these experiments from stocks by both mixed and single genotype mating, allowing for littermate controls as well as maintenance of breeding lines. Weaning was performed between 21 and 28 days old with tail snipping and genotype confirmation at 14 days old. Animals were maintained on individually ventilated racks in a room maintained on a 12 : 12 light : dark cycle with time on at 06.oo and off at 18.00 and fed ad libitum. Animals were 77–113 days old for both genotypes (average ages per genotype 113 days old B6, 103 days old *Fmr1* KO). Numbers represent both sections from (number of animals), B6 males 106 images (3 animals), B6 females 77 images (3 animals), *Fmr1* KO males 115 images (5 animals), *Fmr1* KO females 49 images (3 animals). The variability in the number of images and animals is due to blinding of the genotypes and subsequent genotyping of animals used in the study. Animals were genotyped using Transnetyx (Cordova, TN).

### 2.3. Tissue preparation

Tissue preparation was performed similar to previous work [21,37]. Mice were anaesthetized with pentobarbital (120 mg kg^−1^ body weight) and transcardially perfused with ice-cold phosphate-buffered saline (PBS; 137 mM NaCl, 2.7 mM KCl, 1.76 mM KH2PO4, 10 mM Na_2_HPO_4_ Sigma-Aldrich, St Louis, MO), followed by perfusion with 4% paraformaldehyde (PFA). Following perfusion, animals were decapitated, brainstems removed and post-fixed in 4% PFA overnight at 4°C. Brainstems were then washed for 10 min three times in PBS and covered in 4% agarose. Fixed brains were then sliced into 50 μm coronal sections with a Vibratome (Leica VT1000s, Nussloch, Germany) that included the MNTB.

### 2.4. Immunofluorescence

Once the brainstem was sliced into roughly 10 sections/animal, free-floating slices were blocked in antibody media (AB media: 0.2 M phosphate buffer (PB; 0.2 M KH_2_PO_4_, 0.2 M Na_2_HPO_4_), 150 mM NaCl, 3 mM Triton-X, 1% bovine serum albumin (BSA)) and 5% normal goat serum (NGS) for 30 min at room temperature on an orbital shaker. Following blocking, sections were incubated with an anti-PMPCB polyclonal antibody raised in rabbit (Proteintech cat. no. 16064-1-AP, RRID:AB_2167122) and used at a 1 :1 000 dilution, with an anti-TOMM20 (Abcam cat. no. ab56783, RRID: AB_945896) monoclonal antibody raised in mouse and used at a 1 : 5000 dilution, and 1% NGS in AB media without Triton-X overnight at 4°C on a rotating shaker. Slices were then washed (3 × 10 min) in PBS (137 mM NaCl, 2.7 mM KCl, 1.8 mM KH_2_PO_4_, 10 mM Na_2_HPO_4_) followed by secondary antibody goat anti-rabbit Alexa 647 (Invitrogen, Carlsbad, CA; H + L, cross adsorbed cat. no. A21244) used at a 1 : 5000 dilution (RRID: AB_2535812), goat anti-mouse Alexa 488 (Invitrogen, Carlsbad, CA; H + L, highly cross adsorbed, cat. no. A32723TR) used at a 1 : 5000 dilution (RRID: AB_2633275), and 1% NGS in AB media without Triton-X for 1 h at room temperature on an orbital shaker. Slices were then washed in phosphate buffer (PB; 0.2M KH_2_PO_4_ and mounted on glass slides with Fluoromount-G (SouthernBiotech, cat. no. 0100-01, Birmingham, AL) and coverslipped (1.5 coverslip). At least 24 h later, slides were taken to a Zeiss Airyscan at Oklahoma State to identify MNTB on slides that were circled on the reverse side of the slide for identification in Rennes. Animal identification was then blinded and slides were shipped cold overnight for imaging in Rennes.

### 2.5. Antibody characterization

**Figure 1.**
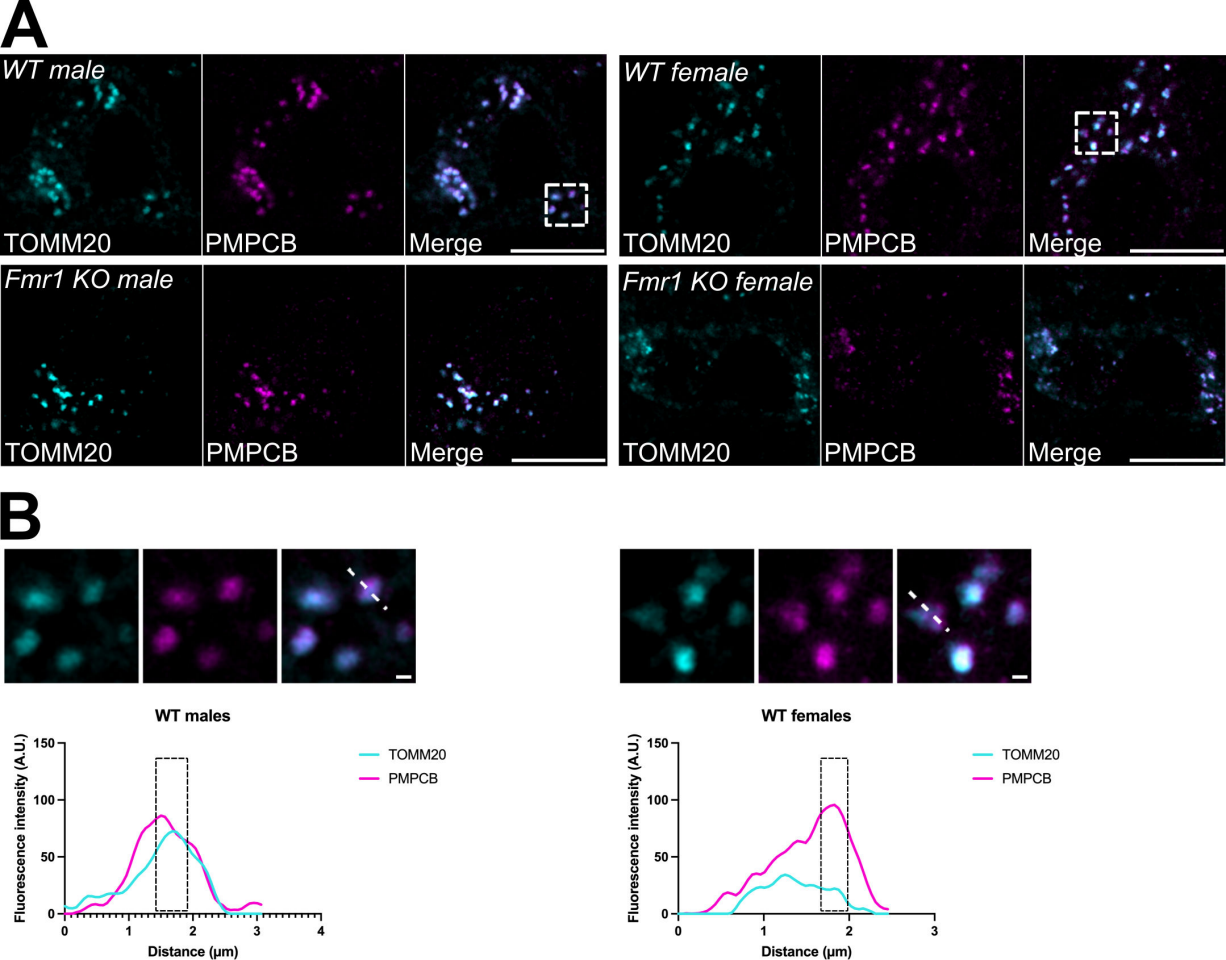
(A) Representative fluorescent micrographs of MNTB neurons co-stained with antibodies against the OMM marker TOMM20 (pseudocolour cyan) and the IMM/matrix marker PMPCB (pseudocolour magenta), in both male and female wild-type (WT) or *Fmr1* KO mice. TOMM20 and PMPCB were merged to show marker colocalization. Scale bar: 10 µm. Dotted area: magnified region of interest (ROI) used for line analyses as in (B). (B) Magnification from insets in (A) and line analyses performed on WT male and female mice, showing the separation between the TOMM20 and PMPCB fluorescent signals over distance. Scale bar: 1 µm. Black dashed areas in the graph: representative regions where a peak in the intensity of one mitochondrial signal corresponds to a lowered intensity in the other channel. A.U.: arbitrary units.

The anti-TOMM20 primary antibody was used for the detection of the outer mitochondrial membrane (OMM), while the anti-PMPCB primary antibody was used to label the IMM/matrix compartment. To visualize these primary antibodies, two complementary fluorescent-conjugated secondaries were used. The goat anti-mouse Alexa 488 was used to detect the anti-TOMM20 primary antibody, and the goat anti-rabbit Alexa 647 was used to detect the anti-PMPCB primary antibody. The anti-PMPCB antibody (Proteintech cat. no. 16064-1-AP, RRID:AB_2167122) is a polyclonal antibody that is specific to the PMPCB fusion protein Ag8937, which corresponds to a 1-349 amino acid sequence encoded by the Mitochondrial Processing Peptidase beta gene (Genebank identifier: BC010398.1). This antibody was previously validated for *PMPCB* knockdown efficiency [38,39]. The anti-TOMM20 primary antibody is a mouse monoclonal antibody specific to the human TOMM20 at amino acids 1-145. TOMM20 staining was validated through its partial co-localization with PMPCB (figure 1B,C) in addition to a consistent staining pattern with other citations using the same antibody in the brain [40].

### 2.6. Imaging

All slides were imaged blinded to genotypes. Multicolour images were acquired as individual optical sections in *xy*, with a Leica SP5 inverted confocal microscope (Leica) driven by the Leica Acquisition Suite (LAS) software, a 63X (N.A. 1.4; Airy units: 1.0) oil immersion objective. The excitation/emission wavelengths for Alexa 488 were 488 and 525/50 nm, respectively; for Alexa 647 were 633 and 650/20 nm, respectively. The 488 nm laser power was set at 40%, and the 633 nm laser power was set at 20% throughout conditions. For visualization purposes only, representative images were deconvolved using the Fiji DeconvolutionLab plugin and the Tikhonov–Miller algorithm [41], with built-in settings used throughout conditions. Brightness and contrast were adjusted and applied to the entire image.

### 2.7. Quantification of images

Raw images were used for quantification throughout the article. To define the smallest size corresponding to a mitochondrial object, a line analysis was performed prior to quantifications. All objects smaller than 15 px (0.7 µm) could not be unambiguously attributed to mitochondrial objects only. Therefore, all objects with a size ≤15 px were considered as background and discarded, while all objects with a size ≥15 px could be unambiguously attributed to mitochondrial objects and were then used for image analyses. The maximal number of pixels per object was set at 45 px (2.2 µm), which corresponded to the maximal size of individual mitochondria throughout conditions following Kalman filtering as previously done [42]. This allowed a count of aggregated-like structures as individual organelles. Fluorescence colocalization (Mander’s M1 and M2 coefficients) between TOMM20 and PMPCB was calculated with the JaCoP plugin [43] of the Fiji software (NIH), with the built-in automatic threshold mask applied to the confocal images. Colocalization analyses were controlled with the JaCoP built-in Costes’ automatic threshold and Costes’ randomization features [43]. Mitochondrial length and branching were calculated on the PMPCB-specific staining, using previously validated procedures to extract aspect ratio and form factor coefficients as indicators of mitochondrial morphology [42,44]. Briefly, aspect ratio is the ratio between the major and minor axes of each mitochondrial object, whereas form factor is calculated by performing the inverse of circularity (1/circularity). For each mitochondrial object, the aspect ratio and circularity were extracted thanks to the Fiji feature ‘shape descriptors’, and then the form factor was calculated for each circularity value. This procedure also provides the overall number of PMPCB-positive mitochondrial objects, which were indicated as mitochondrial mass.

### 2.8. Statistical analysis

Graphs were generated in R [45] and ggplot2 [46]. Linear mixed effect models were performed to control for repeated measures within animals similar to previous work [24,28,47] (R package lme4, [48]). Genotype and sex were considered fixed effects with animal as a random effect. All results indicated a significant effect of interaction; therefore, estimated marginal means (emmeans [49]) were used to make pairwise comparisons between genotype and sex. A Tukey multiple comparison correction is included in these contrasts using emmeans. For all tests, alpha was 0.05. The results of each test were plotted as violin plots showing the entire data distribution with lines of dots indicating data from individual animals. Significant and non-significant comparisons, together with their respective *p*-values, are indicated in each corresponding figure panel. The factor analysis of mixed data (FAMD) was performed with R studio v. 2024.04.0-735, and using the functions associated with the *FactoMineR* and *Factoextra* libraries, including the Fviz functions. Genotype and sex were used as qualitative variables. Mander’s M2 coefficient and mitochondrial numbers were used as quantitative variables, with *n* = 90 MNTB neurons from three WT males, 60 MNTB neurons from three WT females, 120 MNTB neurons from five *Fmr1* KO males and 90 MNTB neurons from three *Fmr1* KO female mice. The correlation circle was built using quantitative variables only, while the FAMD maps were built using quantitative and qualitative variables. Quantitative and qualitative variables were normalized during the analysis in order to balance the influence of each set of variables.

## 3. Results

### 3.1. Mitochondrial morphology of the medial nucleus of the trapezoid body cells is not affected in Fragile X Syndrome mice

Previous reports showed altered mitochondrial morphologies in FXS models. In fibroblasts generated from FXS patients, mitochondria display a doughnut-like shape and have an increased number of cristae when compared with controls [4]. A decreased endoplasmic reticulum (ER)–mitochondria distance is also observable in the hippocampus of *Fmr1* KO mice [50], and it is known that mitochondrial morphology depends on the proximity of the organelles to the ER [51].

**Figure 2.**
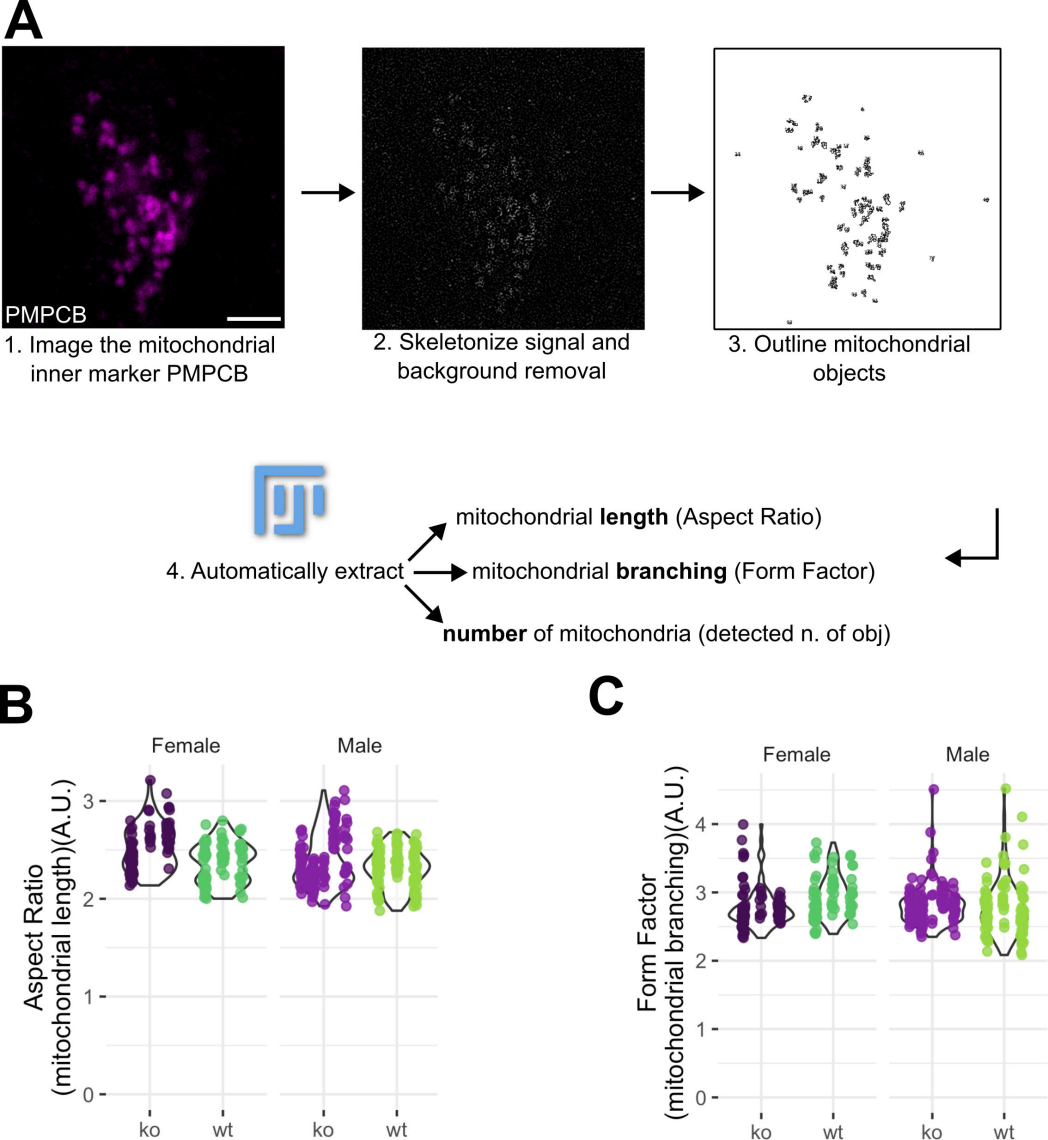
(A) Fiji-based image analysis pipeline to automatically extract mitochondrial length, branching and the overall number of mitochondria from confocal images. Once PMPCB-positive, *xy* individual optical sections were obtained as in figure 1 (representative micrograph, pseudocolour magenta) (1), the signal is skeletonized (2) to obtain mitochondrial objects (3). Scale bar: 10 µm. For every object, the aspect ratio and the form factor were calculated following [42,44]. The overall number of PMPCB-positive objects of ≥15 px was also extracted. (B) Violin plots of aspect ratio and (C) form factor analyses in male and female mice, either wild-type (WT) or KO for *Fmr1*. All analyses were performed as in (A) *n* = 90 MNTB neurons from three WT males, 60 MNTB neurons from three WT females, 120 MNTB neurons from five *Fmr1* KO males and 90 MNTB neurons from three *Fmr1* KO female mice. Data extend from min to max. Each vertical line of dots represents data from individual animals with individual *n* measurements shown as dots in each condition (dark purple Fmr1 KO females, darker green B6 wild-type females, lighter purple Fmr1 KO males and lighter green B6 wild-type males). A.U.: arbitrary units.

Since FXS patients and mouse models show alterations to their auditory system [19], we hypothesized that mitochondrial morphology alterations are also present in *Fmr1* KO mice MNTB neurons. To this end, we co-immunostained MNTB sections of wild-type (B6) and *Fmr1* KO mice with the OMM marker TOMM20, and with the matrix marker PMPCB. These markers were chosen because of their high abundance, their specificity in cells and in tissues, and because they allow us to distinguish the most external and the most internal part of the organelles. MNTB neurons from male and female wild-type and KO mice were then imaged with confocal microscopy (figure 1A). As expected, line analyses showed that the TOMM20 and PMPCB signals are not perfectly colocalizing (figure 1B). This corroborates the power of our imaging approach to distinguish two signals known to be at a distance of approximately 20−50 nm, depending on the cell type [52]. Then, we used the PMPCB-specific signal to calculate mitochondrial aspect ratio and form factor (figure 2). These two parameters report on organelle length and branching, respectively [42]. We observed that mitochondrial length in MNTB neurons is similar between male and female wild-type mice. In addition, male and female *Fmr1* KO mitochondria were no longer than their wild-type counterparts, despite a small tendency towards longer organelles in *Fmr1* KO mice (figure 2A,B). Lastly, mitochondrial branching showed no difference between sexes or genotypes (figure 2A,C).

Overall, confocal imaging of MNTB neurons shows no differences in mitochondrial length and interconnectivity in the presence or absence of *Fmr1*. Since mitochondrial morphology is not globally affected in this neuronal population, we then asked whether organelles are intact or not in FXS mice.

### 3.2. Mitochondrial membrane integrity in the medial nucleus of the trapezoid body cells is decreased in female Fragile X Syndrome mice

In humans, FXS affects males roughly two times more than females both in prevalence and clinical manifestation [53]. Both mitochondrial function and auditory brainstem physiology show sex-specific differences in FXS astrocyte culture and mouse models [12,25]. Indeed, mitochondrial structure and function have been linked to sex differences in several pathologies [14] and it is increasingly accepted that mitochondria are sexually dimorphic in metabolism and cell death [54]. The integrity of the IMM and OMM, respectively, are critical to the function of mitochondria. When the integrity of the membranes is compromised, there are alterations to the membrane potential with depletion of ATP generation capacity and energy alterations [55,56]. A loss of membrane integrity is also key to initiating mitochondrial turnover pathways by mitophagy [57–59].

**Figure 3.**
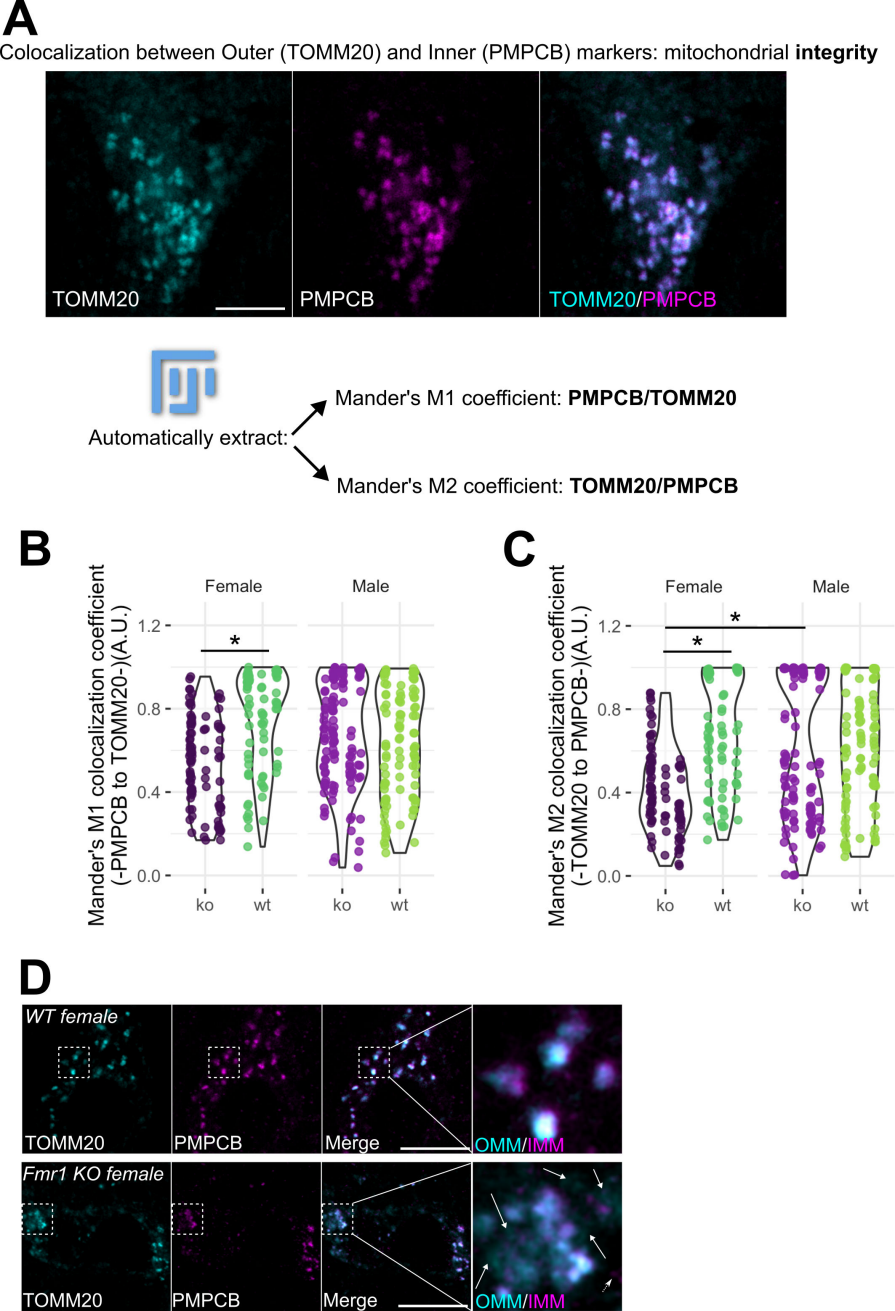
(A) Representative fluorescent micrographs and Fiji-based image analysis pipeline to automatically extract mitochondrial integrity from confocal images using the JACoP plugin [43]. Once TOMM20- (pseudocolour cyan) and PMPCB- (pseudocolour magenta) positive *xy* individual optical sections were obtained as in figure 1, Mander’s M1 and M2 coefficients were calculated using the JACoP plugin. The performance of these calculations was controlled with Costes’ automatic threshold, randomization and with a cytofluorogram directly with JACoP built-in solutions, and this for every pair of images analysed. Scale bar: 10 µm. (B) Violin plots of Mander’s M1 and (C) of Mander’s M2 coefficients in male and female mice, either wild-type (WT) or KO for *Fmr1*. All analyses were performed as in (A) *n* = 90 MNTB neurons from three WT males, 60 MNTB neurons from three WT females, 120 MNTB neurons from five *Fmr1* KO males and 90 MNTB neurons from three *Fmr1* KO female mice. Data extend from min to max. Individual *n* measurements are shown as dots in each condition and animals represented by lines of dots (dark purple Fmr1 KO females, darker green B6 wild-type females, lighter purple Fmr1 KO males and lighter green B6 wild-type males). Values span from 0 to 1, where 0 indicates no colocalization and 1 indicates perfect colocalization. *p ≤ 0.05; all other comparisons were not significant. (D) Representative fluorescent micrographs of MNTB neurons co-stained with antibodies against the OMM marker TOMM20 (pseudocolour cyan) and the IMM/matrix marker PMPCB (pseudocolour magenta), in wild-type (WT) or *Fmr1* KO female mice. TOMM20 and PMPCB were merged to show partial marker overlap. Dotted area: magnified region of interest (ROI) to illustrate membrane integrity loss in *Fmr1* KO mice compared with wild-type. Arrows show TOMM20-positive, but PMPCB-negative mitochondria. The dotted arrow shows PMPCB-positive, but TOMM20-negative mitochondria. Scale bar: 10 µm. A.U.: arbitrary units.

To address whether the ratio of the IMM and OMM was compromised in the MNTB of FXS, we calculated both the Mander’s M1 (PMPCB-IMM to TOMM20-OMM) and M2 (TOMM20-OMM to PMPCB-IMM) colocalization coefficients using the stained sections imaged above in male and female FXS mouse MNTB (figure 3A). We found no significant difference between male genotypes in the M1 and M2 coefficients (figure 3B,C). In contrast, female FXS mice showed significant reductions in M1 and M2 coefficients compared with wild-type females, suggesting a decreased mitochondrial membrane integrity (figure 3B–D). Lastly, FXS males showed a higher M2 coefficient and therefore a higher integrity compared with FXS females (figure 3C).

In summary, FXS females show a severe membrane phenotype, with decreased integrity in both IMM and OMM ratios. In MNTB neurons, FMRP loss leads to mitochondria with either OMM- or IMM/matrix-only markers (figure 3D). This might lead to decreased ATP production in the MNTB of FXS females and to an overall loss of mitochondria in these conditions.

### 3.3. Male Fragile X Syndrome mice show mitochondrial mass loss in the medial nucleus of the trapezoid body

Prior data obtained from fibroblasts of FXS patients indicated that there is either no overall loss of mitochondrial proteins [50] or an increase of specific proteins related to mitochondrial respiration, such as the ATPase inhibitor IF1, cyclophilin D (CyPD) or the ATP synthase OSCP subunit [4]. However, MNTB mitochondria showed a loss of membrane integrity in FXS female mice (figure 3B–D). We reasoned that this could be the result of an overall loss of organelle mass under these conditions. In addition, work in cell models also showed a link between FMRP and the efficient clearance of defective mitochondria by mitophagy [50].

**Figure 4.**
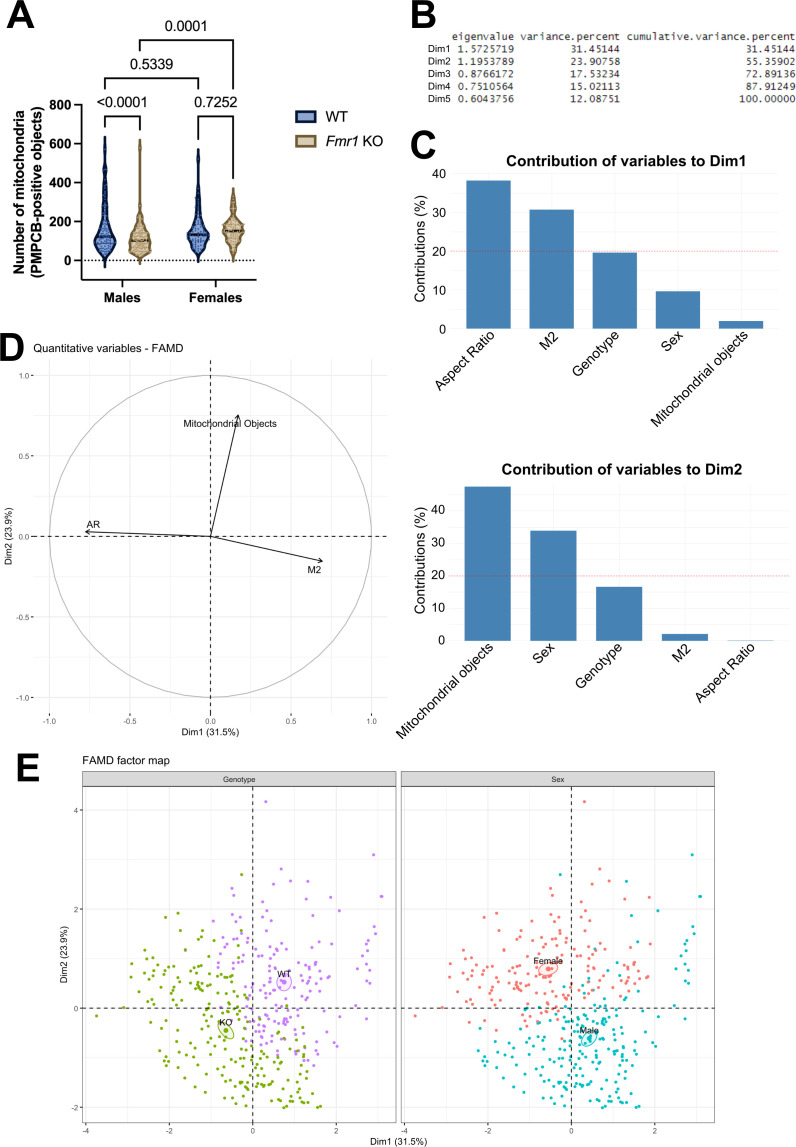
(A) Violin plots of the number of PMPCB-positive mitochondrial objects in male and female mice, either wild-type (WT) or KO for *Fmr1*. All analyses were performed as in figure 2A. *n* = 90 MNTB neurons from three WT males, 60 MNTB neurons from three WT females, 120 MNTB neurons from five *Fmr1* KO males and 90 MNTB neurons from three *Fmr1* KO female mice. Data extend from min to max. Individual *n* measurements are shown as dots in each condition with lines of dots representing animal-level distributions (dark purple Fmr1 KO females, darker green B6 wild-type females, lighter purple Fmr1 KO males and lighter green B6 wild-type males). A.U.: arbitrary units. (B) Raw and percentage of eigenvalue variance, and percentage of cumulative variance of data dimensions in our datasets. In the cumulative variance, values add up to reach the entire (100%) dataset. (C) Contribution of each indicated variable to the first data dimension (in percentage, upper graph) or to the second data dimension (in percentage, lower graph). The red dashed line indicates the expected average value. (D) The correlation plot between aspect ratio, the number of mitochondrial objects and the Mander’s M2 coefficient across the first and second data dimensions. (E) FAMD factor score plots showing the genotype- and sex-dependent clustering of the entire dataset across the first and second data dimensions. Individual *n* values as in (A) are shown as dots. Pseudocolours green and purple were used to represent the genotype, while pseudocolours blue and red were used to represent the sex.

To address this possibility, we calculated the number of PMPCB-positive organelles, which reflects the mitochondrial mass, across sexes and genotypes using confocal microscopy and image analysis (figure 2A). Our analyses revealed no difference in the number of mitochondrial objects between wild-type and *Fmr1* KO mice (figure 4A). Therefore, our data in MNTB neurons confirm previous reports showing no loss of mitochondria in FXS mice [50].

So far, we observed that mitochondrial integrity was lowered in *Fmr1* KO females, while no significant difference was observed in terms of mitochondrial morphology and number in FXS mice compared with wild-type. Therefore, we asked whether these parameters may still be correlated in MNTB neurons or whether these are independent from each other. To this end, we performed FAMD, a method suitable to analyse mixed datasets. FAMD allows the analysis of quantitative variables as a principal component analysis (PCA), and of qualitative values as a multiple factor analysis (MFA) [60]. In our analyses, the aspect ratio, Mander’s M2 coefficient and mitochondrial number were used as quantitative variables, while sex and genotype were used as qualitative variables. First, we determined that our dataset can be described by five data dimensions, and that the first and the second data dimensions together account for 55% of all the variables needed to illustrate the entire dataset (figure 4B). We then evaluated the contribution of each quantitative and qualitative variable to the two main data dimensions. We observed that the aspect ratio, the Mander’s M2 coefficient and the genotype largely contribute to the first data dimension. In contrast, mitochondrial objects and sex are the main variables representing the second data dimension (figure 4C).

We then explored the correlation between the three most relevant quantitative variables contributing to data dimensionality—mitochondrial length, integrity and number—regardless of sex and genotype. We found that the Mander’s M2 coefficient and the aspect ratio, both contributing to the first data dimension, are anti-correlated (figure 4D). Furthermore, the Mander’s M2 coefficient and mitochondrial objects, which are contributing largely to the second data dimension, are positively correlated (figure 4D). Lastly, we evaluated how genotype (WT versus *Fmr1* KO) and sex (male versus female) are distributed in the two data dimensions, for each MNTB neuron imaged within the dataset. This representation integrates the two qualitative variables across the two main dimensions that are mainly shaped by quantitative variables (figure 4C). According to the FAMD factor maps, MNTB neurons can be segregated into two main groups either in terms of genotype (left score plot) or sex (right score plot) (figure 4E). The overlap between the two clusters was limited. In the left score plot, we observed that mitochondria from KO MNTB neurons show a positive correlation between the aspect ratio (Dim. 1) and the number of mitochondrial objects (Dim. 2), while showing an anti-correlation between the aspect ratio and the Mander’s M2 coefficient (Dim. 1) (figure 4E). When looking at sex-specific effects in the right score plot, we observe that the MNTB neurons with longer, more abundant and less intact mitochondria are those from female mice. Conversely, mitochondria from males have a low amount of mitochondrial objects, a low aspect ratio and a high proportion of intact mitochondria. Overall, FAMD analyses show that mitochondrial morphology, integrity and mass are correlated in MNTB neurons. This, in turn, is correlated with sex and genotype of wild-type and FXS mice.

## 4. Discussion

We provide evidence that mitochondria are altered in MNTB neurons of mice affected with FXS. In FXS mice, these alterations occur at the level of mitochondrial membrane integrity. These findings have significant implications on the function of auditory mitochondria and could explain some of the auditory-specific phenotypes seen in mouse models and people with FXS due to the high energetic demands of this region. Interestingly, there were no major alterations in terms of mitochondrial morphology and number. This confirms previous reports [50] and strongly suggests that compensatory pathways could be activated to overcome major mitochondrial mass alterations and loss.

As previously described, mitochondrial respiration and ROS production in astrocytes of FXS mice were reported to be sex-specific, supporting a role for sex-specific differences in mitochondrial function in FXS [12]. However, questions remain regarding potential mechanisms underlying sex differences, and comparisons between the current study and others are difficult, for example [12] was performed in isolated primary astrocytes, which were then cultured, permeabilized and processed for *in vitro* analyses. It is also worth considering that astrocytes and MNTB neurons have different physiology and may show intrinsic variability in mitochondrial functions, including metabolism. Isolating and culturing astrocytes may result in these cells adapting their functions *ex vivo*. As a consequence, this may explain why the majority of sex-specific differences were overall modest compared with the current study, and were only present in hypoxic conditions. The sex-specific differences observed in MNTB neurons not only expand the original findings generated in astrocytes to an alternative neuronal population but also show that significant mitochondrial morphology, integrity and mass differences exist at the tissue level. It is important to note that our female mice have two affected X chromosomes, a condition that is very rare in the human population. However, this somewhat derived condition regarding human health is important for comparison since it limits the possibility of variability in heterozygous females, where it is unknown which X is active and allows our findings to apply more broadly to differences that could occur due to sex alone. Lastly, a recent study performed in mice quantified the maternal transmission of an active *Fmr1*-KO allele to be higher than the paternal transmission [61]. This finding may explain why the mitochondrial integrity phenotype is stronger in MNTB neurons from female KO mice than what we observe in males. This also raises the interesting possibility that mitochondrial signalling pathways may be differentially regulated in males and females. In this light, the presence of local chromosome X activation/inactivation mosaicism correlates with the prevalence of specific symptoms [61], and these differences could also be translated at the subcellular level. Although future studies are required to corroborate this possibility, this may pave the way for the integration of sex specificity and the mitochondrial phenotypic variability in therapeutic solutions against FXS.

Despite the importance of the auditory brainstem in terms of energetic demands, for example being able to generate action potentials up to 1000 Hertz [15], few studies have focused on mitochondrial function in this region [16,62–65]. While other studies included both male and female animals, due to the constraints of *in vitro* preparations, or developmental questions, they were all performed at young ages (less than postnatal day 40) where it is unexpected for there to be sex differences and/or it is difficult to discriminate the sexes [16,17,62–65]. To our knowledge, this is the first study to characterize sex differences in auditory brainstem mitochondria, which has important implications for sex differences in sound information. Open questions remain regarding what drives sex differences in this area, if there are cyclical differences in females suggesting estrus-specific effects, or if there are other hormonal contributions to mitochondrial function, which may underlie differences in sound localization in the sexes [28].

Mitochondrial ultrastructure, morphology, respiratory capacity and sensitivity to apoptosis were recently evaluated in cultured fibroblasts from FXS patients [4]. These complementary parameters were used to define the direct impact of FXS-related mutations on mitochondrial functions. In the present study, an integrated imaging pipeline allowed quantification of mitochondrial phenotypes directly on a tissue relevant for the disorder. The pipeline needs to be calibrated to define the minimal size of a mitochondrial-specific object. This parameter is mandatory for executing the entire pipeline, as it allows for distinguishing the organelles from the background. This calibration is rather straightforward when imaging abundant mitochondrial proteins such as PMPCB or TOMM20. Nevertheless, alternative mitochondrial markers may have a higher signal-to-noise ratio, or may be unevenly distributed at the sub-mitochondrial level. This could hamper the calibration of the pipeline and may give rise to different results from those reported here. Despite these considerations, data obtained with this automated pipeline and analysed with FAMD analyses show that three parameters, such as mitochondrial morphology, integrity and mass, are correlated in MNTB neurons. First, the high number of images and of mitochondria contained in each image of the dataset showcased the intercellular variability of mitochondrial phenotypes. Then, FAMD analyses allowed the combination of the intercellular variability with the inter-individual one, leading to the conclusion that mitochondrial morphology, integrity and mass are correlated in a sex- and genotype-related manner. Overall, this demonstrates the power of confocal microscopy to provide robust and quantitative data at the subcellular level, and to uncover correlations among mitochondrial features that could remain unnoticed with less resolutive approaches.

The auditory brainstem provides a unique model to test for mitochondrial changes in an area of the brain that requires some of the most energetic synapses in order to encode sound information. In addition, there are advantages to using anatomical approaches in adult animals to characterize mitochondrial phenotypes such as the ability to correlate changes with auditory phenotypes in FXS such as altered auditory brainstem responses [25] or *in vitro* physiology [22,31,33,66,67] that is typically limited to younger developmental ages. Specifically, these results could be related to decreased amplitude of wave IV (an area that may represent MNTB processing) of the ABR in female *Fmr1* KO mice (compared to female *Fmr1* KO heterozygotes—almost significant compared to wild-type), however, further work directly linking mitochondrial issues to ABR phenotypes would need to be performed to validate this connection. Understanding adult phenotypes is an important step towards testing for efficacy of drug interventions that might target mitochondria in patients who already have FXS. In addition, while powerful for understanding mechanisms, it can be difficult to connect fibroblast or culture studies with biologically relevant systems, like auditory processing.

### 4.1. Future directions

A previous report provided a connection between mitochondrial mass loss and Parkin/PINK1-related mitophagy in FXS models [50]. Our study shows a loss of mitochondrial integrity in MNTB neurons from male FXS mice. Therefore, future analyses in this neuronal population will demonstrate whether specific mitophagy pathways are active. Pathways such as piecemeal mitophagy, a mitochondrial degradation pathway active at basal levels [68], could potentially explain the observed loss of integrity and no overall mitochondrial mass loss. Complementary information could also be obtained with ultrastructural data, where the morphology and the integrity of individual mitochondria are estimated with electron microscopy-based analyses. This approach would also allow for a direct comparison of the ultrastructural changes previously observed in fibroblasts [4] and in MNTB neurons.

Furthermore, determining the mitochondrial energetic capacity in MNTB neurons from FXS mice remains pivotal. This would provide information on how FMRP may impact energy metabolism in a neuronal population with high energy demands [16]. A previous study highlighted the role of FMRP in regulating the number of ER-mitochondria contact sites (ERMCSs) [50]. FMRP-dependent ERMCSs were shown to be key not only for energy metabolism but also for maintaining synaptic structure and function in a *Drosophila* model of FXS through Ca^2+^ dynamics. Therefore, our results pave the way to functionally correlate mitochondrial integrity, the pathways regulating mitochondrial energetic capacity and the molecular actors orchestrating these functions in MNTB neurons.

Our integrative imaging pipeline, which is entirely based on open-source software, could easily be implemented to explore alternative mitochondrial markers. This could allow us to image additional organelle functions or the regulation of mitochondrial signalling pathways. The imaging pipeline could also be adapted to alternative brain areas, or to measure mitochondrial functions in the auditory brainstem of alternative model organisms. The integrated imaging pipeline presented here is currently optimized for images acquired in two dimensions. Therefore, future improvements in the pipeline should determine whether the mitochondrial phenotypes observed in this study are limited to the axial dimension or if they extend to the lateral dimension as well. Presynaptic release requires a lot of energy to maintain neurotransmission, particularly at high-frequency stimulation rates, such as those present at the calyx of Held synapse. Specifically, the calyx of Held presynaptic synapse uses glycolysis to maintain neurotransmission at baseline and low-frequency stimulation [63]. While we do not discriminate in our study between pre- and postsynaptic mitochondria, it is expected based on energetic demands that there may be differences in presynaptic or postsynaptic mitochondrial function and morphology. Due to different energy demands between the pre- and postsynapse, it would be interesting to differentiate between mitochondria at either side of the synapse to show if there are location-specific alterations in mitochondrial form and function. Additionally, higher resolution imaging, such as electron microscopy, would be necessary and can provide finer detail regarding mitochondrial form as well to corroborate our results. Differences in energy demands at rest, low, and high frequency in the presynaptic terminal of FXS mice would also give important insights into the pathways involved, whether glycolytic or due to mitochondrial respiration alterations.

In conclusion, we show here that mitochondrial morphology, mass and integrity are correlated in MNTB neurons across genotype and sex. With our integrated analysis pipeline, we demonstrate that *Fmr1* KO females lose mitochondrial integrity compared with female wild-type animals. Our data not only show that FMRP is key for the maintenance of mitochondrial physiology in MNTB neurons but also highlight sex-specific differences that may arise in this specific neuronal population.

## Data Availability

Data is available on Zenodo [69].
