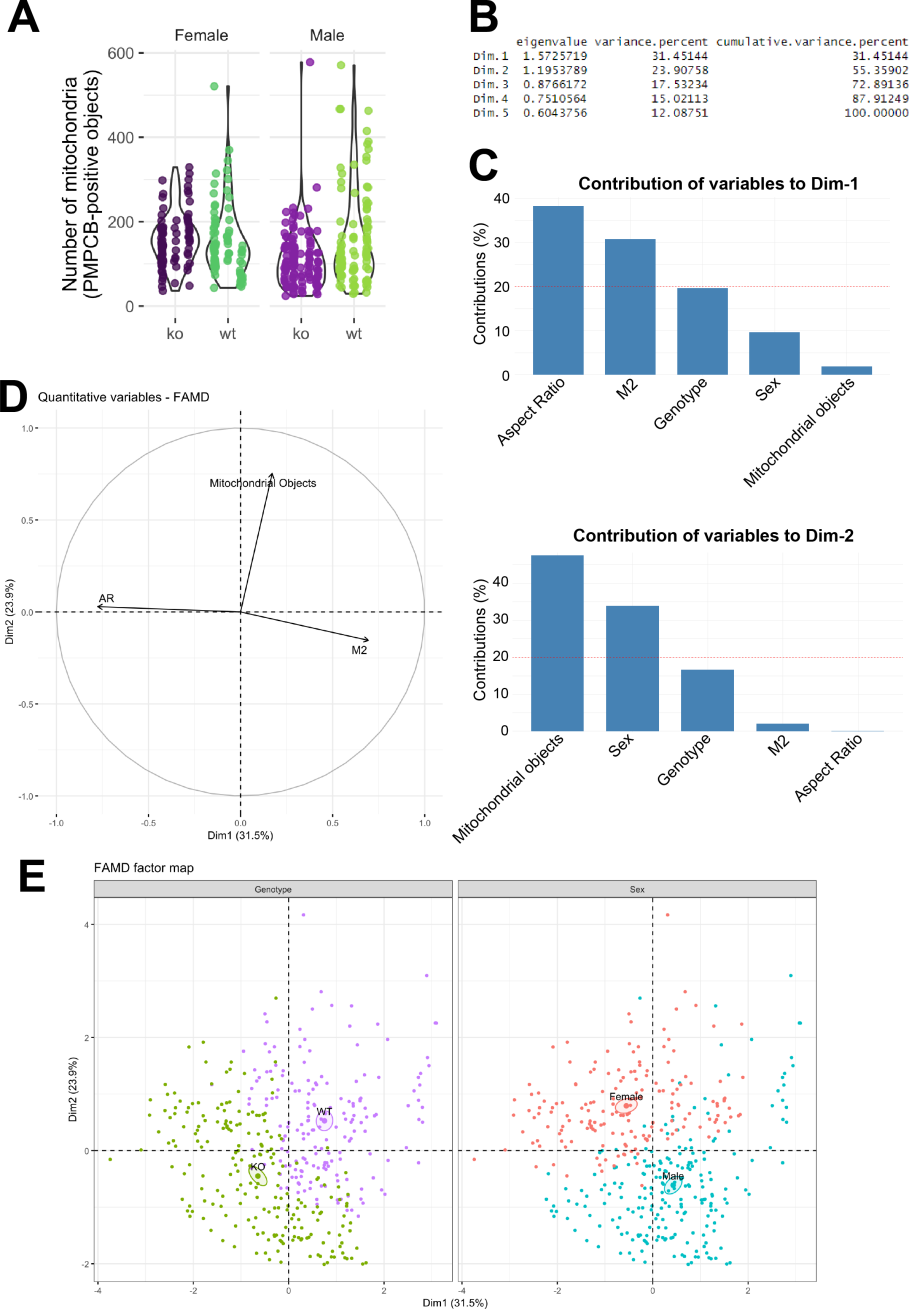# Correction to: ‘Sex-specific loss of mitochondrial membrane integrity in the auditory brainstem of a mouse model of Fragile X Syndrome’ (2024), by Caron *et al*.

**DOI:** 10.1098/rsob.250374

**Published:** 2025-11-26

**Authors:** Claire Caron, Elizabeth Anne McCullagh, Giulia Bertolin

**Affiliations:** ^1^ CNRS, IGDR (Institute of Genetics and Development of Rennes), University of Rennes, Rennes, France; ^2^ Integrative Biology, Oklahoma State University, Stillwater, OK, USA

We replaced figure 4, which was from an older version during the review process, with the updated figure 4 that matches the content of the text.